# Using Internet Search Queries to Assess Public Awareness of the Healthy Cities Approach: A Case Study in Shenzhen, China

**DOI:** 10.3390/ijerph18084264

**Published:** 2021-04-17

**Authors:** Jun Yang, Yutong Zhang, Yixiong Xiao, Shaoqing Shen, Mo Su, Yuqi Bai, Jingbo Zhou, Peng Gong

**Affiliations:** 1 Ministry of Education Key Laboratory for Earth System Modeling, Department of Earth System Science, Tsinghua University, Beijing 100084, China; zhang-yt20@mails.tsinghua.edu.cn (Y.Z.); xiaoyixiong@mail.tsinghua.edu.cn (Y.X.); yuqibai@tsinghua.edu.cn (Y.B.); penggong@tsinghua.edu.cn (P.G.); 2 Tsinghua Urban Institute, Beijing 100084, China; 3 Shenzhen Research Center of Digital City Engineering, Shenzhen Municipal Bureau of Planning and Natural Resource Management, Shenzhen 518034, China; s_s_q@126.com; 4 School of Resource and Environment Science, Wuhan University, Wuhan 430079, China; 2014102050019@whu.edu.cn; 5 Business Intelligence Lab, Baidu Research, Baidu Inc., Beijing 100193, China; zhoujingbo@baidu.com; 6 Department of Earth Sciences, the University of Hong Kong, Hong Kong; 7 Department of Geography, the University of Hong Kong, Hong Kong

**Keywords:** healthy city, health needs, internet search, outreach, public awareness

## Abstract

Cities around the globe are embracing the Healthy Cities approach to address urban health challenges. Public awareness is vital for successfully deploying this approach but is rarely assessed. In this study, we used internet search queries to evaluate the public awareness of the Healthy Cities approach applied in Shenzhen, China. The overall situation at the city level and the intercity variations were both analyzed. Additionally, we explored the factors that might affect the internet search queries of the Healthy Cities approach. Our results showed that the public awareness of the approach in Shenzhen was low. There was a high intercity heterogeneity in terms of interest in the various components of the Healthy Cities approach. However, we did not find a significant effect of the selected demographic, environmental, and health factors on the search queries. Based on our findings, we recommend that the city raise public awareness of healthy cities and take actions tailored to health concerns in different city zones. Our study showed that internet search queries can be a valuable data source for assessing the public awareness of the Healthy Cities approach.

## 1. Introduction

The internet has increasingly linked the world together. It only took 15 years to increase the number of internet users from 1.1 billion in 2005 to 4.0 billion (i.e., 51% of the world population) by 2019 [1]. Billions of people rely on information from the internet to manage their work and lives. Subsequently, the digital footprints left by people when surfing the internet become a gold mine for companies, governments, and researchers to study people’s thoughts and behaviors from the scale of individuals to the entire society.

Internet search queries account for the lion’s share of data generated by internet users. For example, about 3.5 billion search queries per day are made through Google alone [2]. Internet search queries thus provide a large-scale observation of user information-seeking activities and reflect users’ needs and interests instantly [3]. Due to the sheer size and the valuable information in the data, internet search queries have been utilized in various research areas. In health science, the most well-known application of internet search queries is using Google Trends data to detect the pandemics of human influenzas [4]. This seminal work opened the door for a wide range of applications in health science studies after that. These applications include, but are not limited to, detecting epidemiological patterns of infectious diseases and noncommunicable diseases [5,6], revealing the health concerns of the public [7,8], assessing the efficiency of disease prevention and treatment measures [9,10], and measuring the impact of health policies [11].

The World Health Organization has promoted the Healthy Cities program since 1986 to address modern urban health challenges [12]. The principles of this Healthy Settings approach include community participation, partnership, empowerment, and equity [13,14]. Motivating people and getting their active participation in a healthy city project is dependent on their awareness of the approach [15,16]. Interestingly, while public awareness has been emphasized repeatedly in healthy city literature, studies on how the public is aware of healthy cities are scarce. Through an exhaustive search of the major literature databases, we could only identify two relevant studies. One study in Brighton and Hove, United Kingdom found that senior politicians and executives had a lack of awareness of the core values and principles underpinning the Healthy Cities approach after interviewing 24 stakeholders [16]. Another study interviewed 205 stakeholders in five cities in different countries and found two cities had a high awareness (>90%) of the healthy city project among the stakeholders [17]. While the two studies provided helpful information on the public awareness of the Healthy Cities approach, their findings were limited by the small sample size and potential response bias. Therefore, due to the limited number of studies and the limitations of the methods used in existing studies, understanding how well the public is aware of the Healthy Cities approach and its composition remains challenging.

Internet search queries may provide a cost-effective way to evaluate the public awareness of the Healthy Cities approach. For instance, Google Trend data have been used to evaluate the public’s attention on 46 health-related events in the United States in 2017. Ten of those events were found to be impactful health awareness events [18]. Internet search queries were also used to assess the awareness of palliative care in the U.S. and were found to be a novel and freely accessible source of data for monitoring public interest in and awareness of palliative care [19]. More cases on the successful use of internet search queries to assess the public perceptions of events, policies, and management measures can be found outside of the health sciences [20,21,22]. All of those examples point to the potential of using internet search queries to evaluate the public awareness of healthy cities. Nevertheless, no study has explored this potential so far, and its usefulness is still untested.

In this study, we intend to explore the potential of using internet search queries to assess the public awareness of the Healthy Cities approach through a case study in Shenzhen, China. Shenzhen has gone through rapid urbanization in the past four decades. It has changed from a fishing village to the most prominent technology center in China. Its technology-savvy residents are well-connected to the internet. China started a small-scale pilot healthy city program in 1994. However, the program kept a low profile until 2016, when the central government initiated a national program to promote healthy cities. The Shenzhen municipal government set a plan to convert the city into a healthy city in 2019. Since the Healthy Cities approach is relatively new to Shenzhen’s residents, the interest to learn more about the approach should be strong. These combinations made the city an ideal place to conduct the case study. Specifically, we had the following objectives for this study: (1) to reveal the spatiotemporal patterns of search queries on the information relevant to the Healthy Cities approach adopted in Shenzhen and (2) to explore how the demography, environmental conditions, health services, and health status of an area affect the search queries made by the public. Several assumptions were tested using those data: (1) places with higher population densities may have more people interested in health issues, (2) places with a higher percentage of people over 60 years old will make fewer queries, as seniors are less technology-savvy than young people, (3) people living in a better environment may be less worried about the environment’s health risk factors and conduct less queries, (4) the residents’ health status may influence the queries on the Healthy Cities approach, and (5) places with more health institutions will have more search queries, because health workers make the most search queries concerning the Healthy Cities approach.

## 2. Materials and Methods

### 2.1. Study Area

Shenzhen is a megacity in South China. The administrative area of Shenzhen is 1997.47 km^2^ roughly between 22°27′–22°52′ N and 113°46′–114°37′ E. Shenzhen consists of 11 districts and 74 subdistricts. Among the districts, the Shenshan Special Cooperation Zone is located in another city but is administered by the Shenzhen government. It is not included in this study. Shenzhen is a highly developed city. By the end of 2018, the built-up area reached 927.96 km^2^, and the urban population reached 13.03 million. It is the first city in China that has an urbanization rate of 100% [23].

### 2.2. Data Collection

A list of the keywords that are most relevant to the Healthy Cities approach adopted in China was first generated using two official documents issued by the National Patriotic Health Campaign Committee (NPHCC): Guiding Opinions on Constructing Healthy Cities and Healthy Townships [24] and the National Healthy City Indicator System (2018 edition) [25]. These two documents form the basis for the local governments to develop their health city programs. The keywords were extracted from the major actions and indicators cited in the documents. These actions and indicators were based on the Healthy Cities approach promoted by the WHO but were tailored to address the health priorities in China’s cities. The NPHCC classified those actions and indicators into five domains. The final list included a total of 60 keywords (Table 1).

The list of keywords was submitted to Baidu, the most prominent web search engine in China. The daily search queries made through Baidu already reached 4 billion in 2013 [26]. Baidu used the list of keywords to retrieve search queries made by Shenzhen’s residents during 1 January 2019 to 31 December 2019. The daily counts of the search queries on each keyword at the resolution of the subdistrict level were made available by Baidu for this study.

Additionally, data on the demographic, environmental, and health conditions at the subdistrict level were collected. For the demographic data, the population density and the population older than 60 were collected. Areas of green spaces were used as an indicator of the built-up environment. These data and essential geographic data, including the administrative boundaries and building layouts, were obtained from the Shenzhen Research Center of Digital City Engineering, Shenzhen Municipal Bureau of Planning, and Natural Resource Management. The mortality due to noncommunicable diseases for 2016–2018 was used as an indicator of the health status of a place. The data were obtained from the Shenzhen Center for Disease Prevention and Control (CDC). Due to the strict regulation on privacy, Shenzhen CDC only provided mortalities grouped into malignant tumors, heart diseases, cerebrovascular diseases, and endocrine system diseases by age classes at the subdistrict level. In addition to the above data, the point of interest (POI) data in 2018 were obtained from Gaode, one of China’s major map service companies. The POIs for hospitals, community health centers, and public health agencies were extracted as an indicator of the health services.

### 2.3. Data Analysis

Data were preprocessed before the formal analysis. The daily search queries on each keyword were normalized using the total number of search queries to generate the standardized search queries at the subdistrict, district, and city levels. The possible influence of the variations in volumes of internet searches was removed through standardization. The percentage of floor areas of residential buildings in 300 m of a green space larger than 1 ha was calculated as the indicator of green space following the method used in an early study [27]. For mortality data, the four groups of disease mortalities were summed and then normalized using the total resident population to estimate the mortality rate of major diseases at the subdistrict level. The POIs of health agencies were examined, and duplicates were removed before the numbers were summed up at the subdistrict level. All data processing works were done using *R* (Version 3.6, *R* Core Team).

The spatiotemporal patterns of the search queries were analyzed at the city, district, and subdistrict levels. At the city level, the number of search queries for each keyword was first ranked to describe the overall popularity of the keywords. The daily number of search queries on all the keywords was tested for a monotonic time trend using the Mann–Kendall test contained in *R* package *funtimes*. The time series of search queries on the keyword “healthy city” was also examined, as it is the most specific keyword of the Healthy Cities approach. Additionally, the Shenzhen municipal government’s major events during the health city project were overlaid on top of the time series. We visually checked whether these events impacted the search queries. An effect was assumed when the peak of the search queries occurred immediately after the event.

The total numbers of search queries on each keyword over a year were compared to show the difference in each district’s interest at the district level. The total number of search queries over a year at each subdistrict was also compared to capture interest at the subdistrict level. Besides, Moran’s *I* values were calculated for the top five keywords to test whether there were spatial clusters for the search queries among the 74 subdistricts. The Moran’s *I* values were calculated using the spatial autocorrelation function in ArcMap (version 10.6, ESRI, Redlands, CA, USA).

The correlation analysis was conducted to explore the association among the number of search queries and environmental, demographic, and health conditions at the subdistrict level. The Shapiro–Wilk test was first performed to examine the normality of the datasets. Since some data did not pass the test, Spearman’s correlation was used in the analysis. If there was a significant correlation, then the ordinary least-squares model was fit to the pair to examine the influence of the factor on the number of search queries. The R package *Hmisc* was used for calculating the Spearman correlation coefficients and testing the significance of the correlations.

## 3. Results

### 3.1. Overall Statistics of Internet Search Queries

Between 1 January 2019 and 31 December 2019, a total of 12.34 billion internet search queries were made through Baidu by Shenzhen’s residents. The search queries on the list of keywords relevant to healthy cities were 1.06 million, about 0.001% of the total search queries.

**Figure 1:**
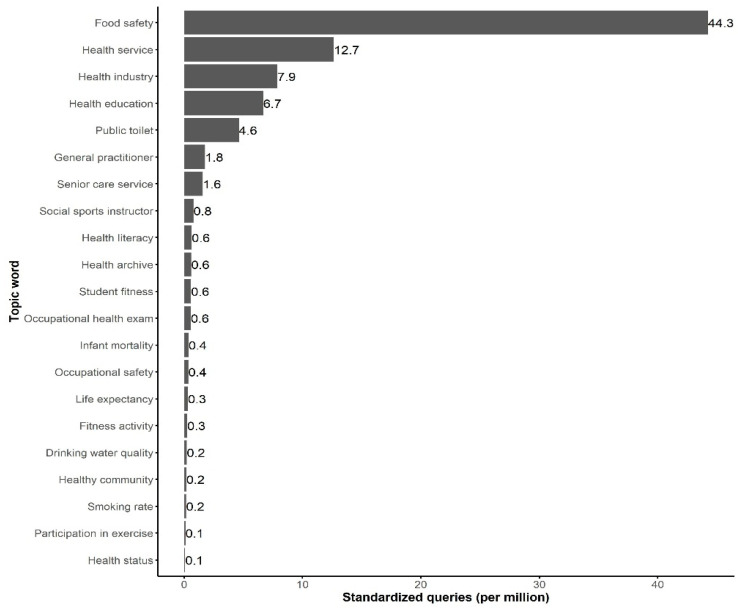
Standardized search queries of the keywords at the city level. Only the keywords with standardized search queries larger than 0.1 per million are shown here.

At the city level, the number of search queries on the keyword “food safety” was the highest, 545,956 counts, while the number of search queries on the keyword “health impact assessment” was the lowest, with only one count (A complete list of the numbers of queries on each keyword can be found in Table S1). After standardizing each keyword’s search queries by the total number of search queries, around 21 out of 60 keywords had values larger than 0.1 per million search queries (Figure 1).

At the district level, the keywords covered by search queries in each district varied substantially. Residents in Futian District made queries on 53 keywords, while residents in Guangming District only searched for 40 keywords (Table 2). Other districts fell in between these two ends, with a mean number of queried keywords of 46.8 (SD = 3.82).

**Figure 2:**
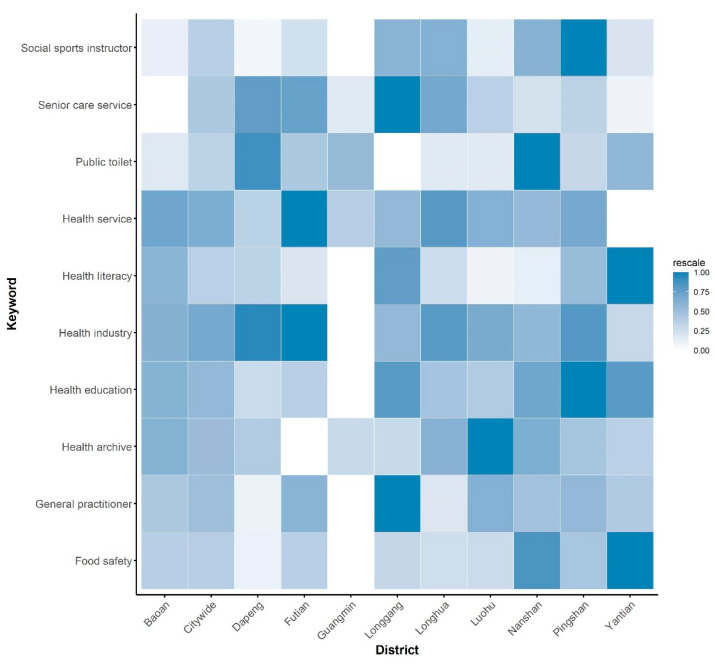
The patterns of the search queries in each district of the top ten keywords queried at the city level. The values were rescaled to 0–1 for comparison.

The pattern of search queries in each district was different. Most districts shared the top keywords with the city, but the magnitudes of the search queries were different (Figure 2).

**Figure 3:**
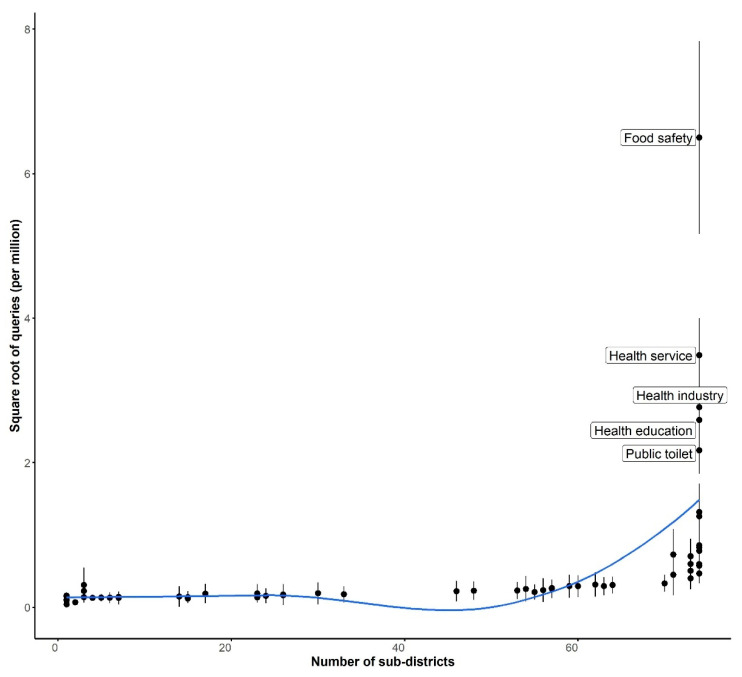
The numbers of search queries on each keyword in the subdistricts plotted against the number of subdistricts where search queries on the keyword were made. The dots represent the mean value, and the length of whiskers indicates the variations among the subdistricts. The top five keywords are labeled.

Most keywords were not queried at the subdistrict level in all subdistricts, and the volumes of search queries were also low. The top keywords were similar to those at the city level. “Food safety” and another nine keywords were queried in every subdistrict (Figure 3).

### 3.2. Spatial Patterns of Search Queries

**Figure 4:**
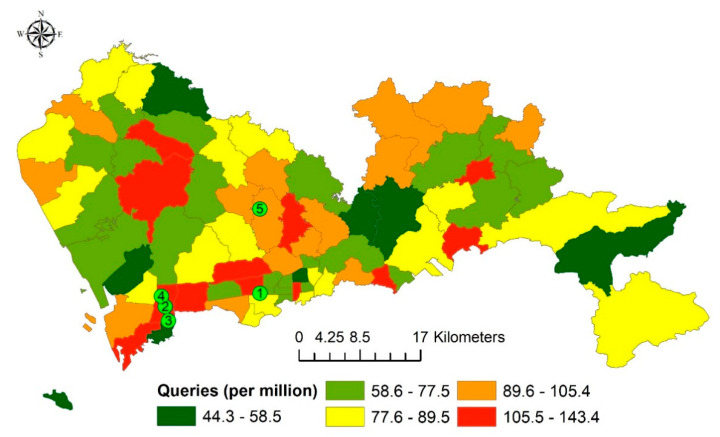
A map showing the sum of standardized search queries in each of the 74 subdistricts. The numbered symbols show the locations of important sites. 1 = Shenzhen Municipal government, 2 = Baidu headquarters, 3 = Ali Center, 4 = Tencent headquarters, and 5 = Huawei headquarters.

The sum of the standardized search queries in each subdistrict was plotted on the map (Figure 4). No obvious spatial patterns could be observed as the subdistricts with high values and low values dispersed among the other subdistricts.

The Moran’s *I* value also indicated no significant clustering patterns for the sum of the standardized search queries and the standardized search queries on the top five keywords among the subdistricts (Table 3).

### 3.3. Temporal Patterns of Search Queries

**Figure 5:**
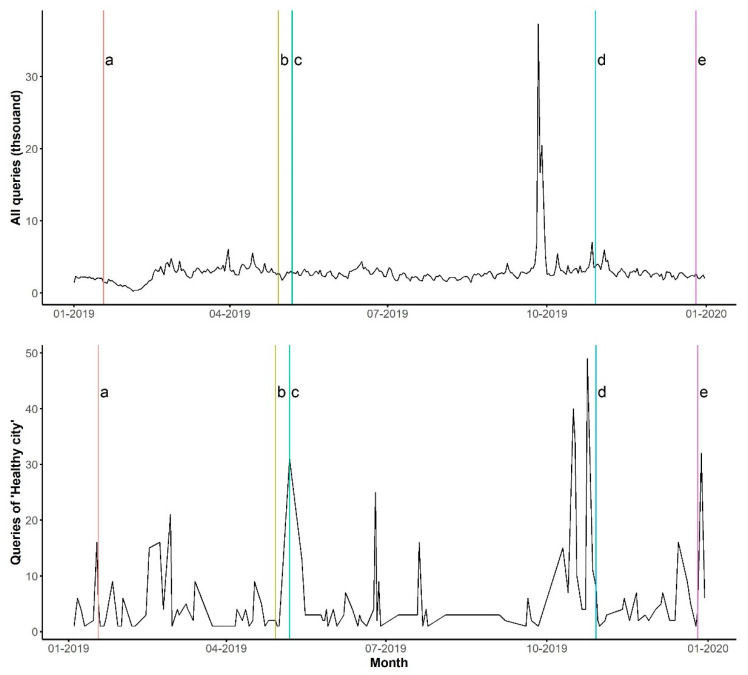
Time series of the search queries on all keywords (upper) and the search queries on the keyword “Healthy city” (bottom) in Shenzhen. Alphabetic letters indicated the major events relevant to the healthy city project in the city.

Since the number of daily search queries on the keywords was not significant, we only examined the time series of the search queries at the city level (Figure 5). The search queries on all the keywords and the search queries on the keyword “Healthy city” did not show any significant time trends (Table 4).

Five major events related to the healthy city project in Shenzhen were examined, together with the search queries. On 20 January, Shenzhen Municipal Health Commission (SMHC) announced the plan to build a healthy city in a leading position internationally (event a in Figure 5). On 29 April, SMHC listed the actions for the healthy city project (event b). On 7 May, SMHC released the Key Points for Health Works in 2019, including the items on healthy cities (event c). On 29 October, SMHC organized the event “Healthy Shenzhen, Healthy city” (event d). On 26 December, Shenzhen launched the Shenzhen Smart Medical International Healthy City project (event e). The figure shows that the peaks of the search queries on all keywords only slightly increased after event d, while the queries on the keyword “Healthy city” increased after events a, b, and e. However, the daily search queries on the keyword “Healthy city” were very low, with the highest number reaching 49 counts. The peak before event d was due to searching for the keyword “food safety” caused by a separated school event on 26 September 2019.

### 3.4. Associations with the Demographic, Environmental, and Health Factors

The number of search queries on all keywords was not significantly associated with the demographic, environmental, and health factors at the subdistrict level (Table 5). The number of search queries on the keyword “Healthy city” was only significantly associated with the number of health institutes at the 95% significance level. However, the number of health institutes could only explain an insignificant portion of the variance in the number of search queries on the keyword “Healthy city” (Adjusted R^2^ = 0.03, *p*-value = 0.11, α = 95%).

## 4. Discussion

### 4.1. Public Awareness of the Healthy Cities Approach

The internet search queries revealed two patterns of public awareness of healthy cities in Shenzhen in 2019: (1) The overall public awareness was low in the city, and (2) public awareness was highly heterogenous at different geographic levels.

The total number of internet search queries on all keywords in a year was 1.06 million for the entire city, less than one-third of the mean daily internet search queries (3.38 million) made in Shenzhen. The queries on the specific term “Healthy city” were only 689 for the entire year. These numbers reflected the lack of awareness or lack of interest among Shenzhen’s residents in the Healthy Cities approach. Considering the program is relatively new to Shenzhen’s residents, the former is more likely than the latter. The lack of public awareness could be due to two reasons. First, the healthy city project is just one of many health promotion programs run by the Shenzhen municipal government, so no extra efforts were committed to promoting the project. European cities treat the Healthy Cities approach as a “whole-system” approach that integrates multidisciplinary actions across the risk factors [28]. In China, the approach is viewed more like one of the many health management approaches. China’s health city program runs parallel with other urban health programs, such as National Hygienic Cities [29] and Chronic disease comprehensive prevention and control demonstration areas [30]. The parallel programs might explain why keywords specific to the Healthy Cities approach received fewer queries than keywords related to other urban health programs, e.g., one for “Health impact assessment” versus 545,956 for “Food safety”. Improving food safety is a component of several health promotion programs.

Second, the main channel used to convey information on the healthy city project was the news on TV and in newspapers and official documents posted on the government website. Occasionally, the government holds public outreach events to promote some aspects of the Healthy Cities approach. For example, the spike in the search queries on all keywords on 26 September 2019 was driven by a public outreach event on food safety in school campuses. The search for the keyword “Food safety” jumped to over 30,000 on that day. However, without a consistent and aggressive information campaign, it is unlikely that the project as a whole will capture the residents’ attention. This point was made clear by the low responses to the meetings and events related to the healthy city project in general.

While the overall awareness was low at the city level, the public awareness was heterogeneous among districts and subdistricts. The keywords that were queried and the total number of queries all varied substantially. The patterns reflected varied health needs at the district and sub-city levels in Shenzhen. Studies in other cities have also shown that urban communities in cities are facing different health challenges. Besides, their capacities for implementing the health city project are also highly varied [31,32]. This tendency was reflected by the various information-seeking behaviors in this study. Higher search queries in some districts and subdistricts than those of others might be due to the simple fact that the former had more health workers than the latter. Therefore, our findings attested to the call that actions taken in a healthy city project should be tailored to meet the health needs of different urban communities in the city [32].

### 4.2. Factors Affecting the Public Queries on the Healthy City Program

We tried to explain the variations in internet search queries by looking into the potential influencing factors. Nevertheless, the demographic, environmental, and health factors examined in this study did not show significant correlations with the number of queries on all keywords. Except for the number of health institutions, those factors also did not correlate significantly with the number of queries on the keyword “Healthy city”. The exception was understandable, as healthy cities in China are primarily administered by the health sector so far [33]. However, the low explanatory power of the number of health institutes on the number of queries indicated stronger influences by other factors.

The failure to identify the factors that significantly affected the internet search queries on the Healthy Cities approach was not surprising. Many human factors can affect internet search behaviors [34,35]. We could not test all of them. Additionally, just as other types of big data, internet search queries are instrumental for making interactions visible but not the hidden laws of causality [36,37]. Since the primary goal of this study is to assess the public awareness of the Healthy Cities approach, i.e., to make residents’ information-seeking behaviors visible, the use of internet search queries is justified. It would be a daunting job to use conventional survey methods to capture the public opinion in a city of 13 million people year-round, if not impossible. Combining Baidu queries with other social media data such as Sina Weibo can capture the public opinion better. However, this possibility can only be realized if the big internet companies grant more access to their data for noncommercial uses.

### 4.3. The Implications of the Current Study

The findings from our study have some important implications for moving forward the healthy city project in Shenzhen. First, the public awareness of healthy cities needs to be improved significantly. A rigorous public outreach program should be implemented as part of the healthy city project. Second, the divergent needs of people in different communities must be addressed with localized plans and actions. This means that bottom-up actions should be adopted, along with the primarily top-down actions taken by the government so far.

## 5. Conclusions

The public awareness of the Healthy Cities approach is key to the success of any healthy city project. Nevertheless, public awareness has rarely been assessed in cities where healthy city projects are deployed. The small sample sizes used in cities that have conducted these assessments may not capture the whole picture of public awareness. In this study, we explored the potential of using internet search queries to assess the public awareness of the Healthy Cities approach. We showed that the data was suitable for this task through a case study in Shenzhen. Shenzhen’s residents had a low awareness of healthy cities. There was also a high intercity heterogeneity in public interests on different components of the Healthy Cities approach. Our assessment results provide helpful information for government agencies to assess the healthy city project’s progress and take remedial measures. The method developed in this study can be applied in other cities in China or other countries to assess their healthy city projects. In the future, a direction that is worth pursuing is to combine internet search queries with other types of big data, such as Twitter and Weibo, to better understand the public perception of the Healthy Cities approach in a city.

## Figures and Tables

**Table 1:** The list of keywords used for retrieving the internet search queries.

Category	Keywords
(1) Healthy environment	(6) Days with good air quality, (7) days with heave air pollution, (8) drinking water quality, (9) green space per capita, (10) harmless disposal of garbage, (11) harmless toilet, (12) public toilet, (13) safety of drinking water sources, (14) sports ground per capita
(2) Healthy society	(15) Beds in senior care facilities, (16) food safety, (17) food sampling inspection, (18) healthy communities, (19) healthy enterprises, (20) health expenditure, (21) healthy schools, (22) occupational health exam, (23) occupational safety, (24) senior care service, (25) social sports instructor
(3) Health service	(26) Basic health insurance reimbursement, (27) children health management; (28) general practitioners, (29) health archive, (30) hospital beds, (31) incidence of malign tumors, (32) maternal and child health management, (33) maternal health management, (34) mental disorder patient management, (35) mental health management, (36) public health workers, (37) the standard of national physique examination,
(4) Healthy population	(38) Disease vector control, (39) health status, (40) incidence of infectious diseases, (41) infant mortality, (42) life expectancy, (43) maternal mortality rate, (44) mortality of children under five, (45) neonatal death, (46) prevalence of high blood pressure, (47) road traffic accident injuries, (48) student fitness
(5) Health literacy	(49) Fitness activity, (50) health education, (51) participation in exercise, (52) smoking rate
Others	(53) Health baseline investigation, (54) health behavior, (55) health equity, (56) health impact assessment, (57) health industry, (58) healthy city, (59) healthy township, (60) pro-health attitude

**Table 2:** Summary statistics of the internet search queries on the keywords at the district level.

District Name	Sum of Standardized Queries (Per Million)	Number of Queried Keywords
Yantian	105.93	42
Nanshan	103.25	47
Pingshan	90.54	45
Futian	87.58	53
Baoan	84.79	50
Longgang	84.27	47
Longhua	81.56	49
Luohu	81.01	49
Dapeng	74.78	46
Guangming	66.45	40

**Table 3:** Moran’s *I* values for the sum of the standardized search queries and the standardized search queries on the top five keywords at the subdistrict level.

Keywords	Moran’s *I* Values	Z-Scores	*p*-Values
All keywords	−0.049	−0.749	0.453
Food safety	−0.064	−1.059	0.289
Health service	0.013	0.555	0.579
Health industry	−0.013	0.018	0.985
Health education	−0.008	0.129	0.898
Public toilet	0.005	0.489	0.624

**Table 4:** Mann–Kendall test results of the times series of the search queries.

Query Type	Mann–Kendall’s τ	*p*-Values	Standardized Queries (Per Million)
Search queries on all keywords	0.09	0.32	85.74
Search queries on the keyword “Healthy city”	−0.02	0.59	0.06

**Table 5:** Spearman correlation coefficients and the *p*-values (values in parenthesis) between the search queries and the demographic, environmental, and health factors at the subdistrict level.

Query Type	Mortality of the Main Diseases (Per Ten Thousand People)	POIs of Health Institutes	Population Density(Person Per km^2^)	% of Senior Residents	% of Residence with Green Spaces in 300 m
Search queries on all keywords	0.08 (0.53)	0.13 (0.30)	−0.02 (0.90)	0.18 (0.15)	−0.07 (0.55)
Search queries on the keyword “Healthy city”	−0.20 (0.17)	0.35 (0.02)	0.14 (0.36)	0.12 (0.44)	−0.08 (0.62)

## Supplementary Materials

The following are available online at https://www.mdpi.com/article/10.3390/ijerph18084264/s1: Table S1: Summary of the internet search queries on the keywords.
